# Monitoring Lipase/Esterase Activity by Stopped Flow in a Sequential Injection Analysis System Using *p*-Nitrophenyl Butyrate

**DOI:** 10.3390/s150202798

**Published:** 2015-01-27

**Authors:** Jorge Pliego, Juan Carlos Mateos, Jorge Rodriguez, Francisco Valero, Mireia Baeza, Ricardo Femat, Rosa Camacho, Georgina Sandoval, Enrique J. Herrera-López

**Affiliations:** 1 Biotecnología Industrial, Centro de Investigación y Asistencia en Tecnología y Diseño del Estado de Jalisco A.C., Avenida Normalistas 800, Colinas de la Normal. C.P. 44270, Guadalajara Jalisco, Mexico; E-Mails: j.pliego.sandoval@zoho.com (J.P.); jcmateos@ciatej.mx (J.C.M.); jrodriguez@ciatej.mx (J.R.); rcamacho@ciatej.mx (R.C.); gsandoval@ciatej.mx (G.S.); 2 Departament d'Enginyeria Química, Escola d'Enginyeria, Universitat Autònoma de Barcelona, 08193 Bellaterra, Barcelona, Spain; E-Mail: francisco.valero@uab.cat; 3 Departament de Química, Facultat de Ciències, Edifici C-Nord, Universitat Autònoma de Barcelona, 08193 Bellaterra, Barcelona, Spain; E-Mail: MariaDelMar.Baeza@uab.cat; 4 Grupo de Biodinámica y Sistemas Alineales, División de Matemáticas Aplicadas, Instituto Potosino de Investigación Científica y Tecnológica. A.C. Camino a la Presa San José 2055, Lomas 4 Sección, C.P. 78216, San Luis Potosí S.L.P., Mexico; E-Mail: rfemat@ipicyt.edu.mx

**Keywords:** monitoring, lipase/esterase activity, sequential injection analysis, stopped flow, *p*-nitrophenyl esters

## Abstract

Lipases and esterases are biocatalysts used at the laboratory and industrial level. To obtain the maximum yield in a bioprocess, it is important to measure key variables, such as enzymatic activity. The conventional method for monitoring hydrolytic activity is to take out a sample from the bioreactor to be analyzed off-line at the laboratory. The disadvantage of this approach is the long time required to recover the information from the process, hindering the possibility to develop control systems. New strategies to monitor lipase/esterase activity are necessary. In this context and in the first approach, we proposed a lab-made sequential injection analysis system to analyze off-line samples from shake flasks. Lipase/esterase activity was determined using *p*-nitrophenyl butyrate as the substrate. The sequential injection analysis allowed us to measure the hydrolytic activity from a sample without dilution in a linear range from 0.05–1.60 U/mL, with the capability to reach sample dilutions up to 1000 times, a sampling frequency of five samples/h, with a kinetic reaction of 5 min and a relative standard deviation of 8.75%. The results are promising to monitor lipase/esterase activity in real time, in which optimization and control strategies can be designed.

## 1. Introduction

During the last few years, the interest in enzymes has increased considerably, since they have important advantages with respect to conventional chemical processes [1]. Lipases (E.C. 3.1.1.3) and esterases (E.C. 3.1.1.1) constitute two of the most important biocatalysts for biotechnological applications. Lipases preferably hydrolyze insoluble triglycerides, while esterases usually hydrolyze water-soluble esters. Since lipases and esterases act in a wide range of substrates, they have many applications [2], such as: degradation of fats and oils through detergents [3], paper bleaching [4], bio-diesel and bio-lubricants [5], the food industry [6] and the resolution of racemic mixtures in drug production [7].

Lipases and esterases are often produced in submerged fermentation by many microorganisms. The most important parameter to be measured in enzyme production is the lipase/esterase activity, defined as: the amount of enzyme that catalyzes the conversion of one μmol of substrate per minute. The hydrolytic activity of lipases and esterases can be determined by different methods, such as: titrimetry, spectroscopy, chromatography, turbidimetry, conductometry, immunochemistry, microscopy and biosensors, among others [8,9]. The standard assay to measure the lipase/esterase hydrolytic activity is the pH-stat technique; however, this approach is time consuming and generates many wastes. To increase the efficiency in measuring the enzymatic activity, spectrophotometric methods employing chromogenic substrates, such as *p*-nitrophenyl and resorufin esters, have been used [10]. The hydrolysis of *p*-nitrophenyl butyrate releases a chromophore (*p*-nitrophenolate), which can be measured spectrophotometrically at 415 nm and at different time intervals [11–13]. This spectrophotometric method is often performed in the laboratory as an off-line analysis, in which a sample must be taken periodically from a bioreactor and analyzed afterward; consequently, the resulting information is not readily available in a computer for on-line monitoring and control of the bioprocess.

Recently, the repetitive and time-consuming activities often performed in analytical chemistry have decreased thanks to the advances in automated microfluidic platforms. Flow injection analysis (FIA) is a set of flow-based techniques, that over the last 30 years has brought speed, automation, miniaturization and low cost, while meeting the highest quality standards required in analytical laboratories. The FIA technique is based on injecting a sample into a unidirectional flowing carrier stream in which reagents are added at confluence points. In this way, the concentration gradient is formed by the dispersion of the sample zone alone. The transient signal reflects the gradient of the sample zone, as it passes through the detector [14]. Flow injection analysis systems can be used to monitor bioprocesses on-line, since it is possible to mix a sample with multiple reagents, including substrates that have low miscibility in water [15]. An FIA system was used to detect glucose and acetate in an *E. coli* fermentation [16]. In other report, an FIA system was developed to measure on-line lipase activity. The turbidity change of the triolein emulsion was measured by a spectrophotometer at 340 nm [17]. Triacylglycerol and glycerol in serum were determined by an FIA system. An enzymatic reaction in a capillary was followed by electrochemical detection. The linear working range for glycerol was from 10^−4^ to 10^−2^ M and from 10^−3^ to 10^−2^ M for triacylglycerol [18]. An automatic system to determine lipase activity was developed using as a substrate 1,2-dioleoylglycerol. The lipase activity was measured from the increase in absorbance at 340 nm in a coupled enzymatic reaction [19]. A potentiometric FIA titration system for monitoring lipase activity was performed using a commercial CaLB enzyme. Triacetin and tributyrin were used as substrates. The detection limit of the enzyme activity was determined in the region 0.035–0.07 U/mL with 15 min of reaction [20].

Sequential injection analysis (SIA) has emerged as the improvement of the FIA system, in which additional flow techniques, such as multi-commutation, multi-syringe and multi-pumping, can be performed. The SIA system operates only during programmed time intervals [21,22], reducing the wear and maintenance of the components [23,24] and saving reagents, since non-continuous consumption is performed. A key component of an SIA system is the multiposition valve that can perform different determinations without the need for the reconfiguration of the equipment [25]. In SIA, a sample-reagent mixture can be serially processed in the different modules connected to the selection valve, by means of repetitive aspiration and injection steps during a specific time [26]. An additional advantage of SIA systems is the possibility of implementing the “stopped flow” approach, which consists of maintaining a sample-reagent mixture in the holding coil (SHC) or in the flow cell (SFC), heated at a high temperature. The readout in SHC mode resembles the flow injection response, since the reaction product flows uninterruptedly through a flow cell. High sensitivity SIA is performed in SFC mode, when the reaction product is arrested in the flow cell, yielding a reaction rate curve. The advantage of the SHC protocol is speed and simplicity, while the SFC protocol offers higher sensitivity and is free from the influence of the refractive index and the background color interference [15,27–29].

An SIA system for monitoring lipase activity produced by *Candida rugosa* using 1,2-*O*-dilauryl-rac-glycero-3-glutaric acid-(6-methylresorufin)-ester as the substrate was designed [30]. This method uses an expensive substrate; in addition, the system could not follow an enzymatic reaction in real time, consequently with the possibility to underestimate or overestimate the lipase activity. Recently, an SIA system was used to monitor pancreatic human lipase with a detection limit of 0.068–3.849 mg/L through a heterogeneous immunoassay using reflectometric interference spectroscopy and total internal reflection fluorescence [31].

FIA and SIA systems have been used to determine lipase/esterase activity in bioprocesses; however, there are still challenges for finding more efficient substrates and monitoring strategies for determining on-line lipase/esterase activity. In this context and in the first instance, we propose to design a lab-made SIA system to analyze lipase/esterase activity using *p*-nitrophenyl butyrate as the substrate, from samples obtained from a shake flask. In the next stage, the SIA system will be coupled to a bioreactor to monitor lipase activity in real time, in which optimization and control strategies can be designed in the near future.

## 2. Materials and Methods

### 2.1. Reagents and Enzymes

Monobasic and dibasic potassium phosphate, magnesium sulfate, *p*-nitrophenyl butyrate, 4-(1,1,3,3-tetramethylbutyl) phenyl-polyethylene glycol (Triton X-100), *p*-nitrophenol, 2-methyl-2-butanol, yeast extract, bacteriological peptone, glucose, agar, olive oil and Tween 80 were purchased from Sigma Aldrich-Fluka (Toluca, Mexico) and local suppliers. The commercial *Candida antarctica* B lipase (CaLB) from Novozyme was used to validate the first measurements in the SIA system.

### 2.2. Yeast Strain, Inoculum Preparation and Fermentation Conditions

The yeast *Yarrowia lipolytica* ATCC 9773 was used as a case study for the lipase production. The strain was stored at 4 °Cand subcultured every month for maintenance. The preinoculum was grown in an assay tube with 5 mL of YPD medium (yeast extract 1% (w/v), bacteriological peptone 2% (w/v), glucose 2% (w/v)) and incubated for 24 h on a rotary shaker at 250 rpm and 30 ^°^C. The inoculum was grown in 25 mL of YPD medium in Erlenmeyer flasks and incubated with the same conditions as the preinoculum. The fermentation experiments were carried out in duplicate in Erlenmeyer flasks of 500 mL with 100 mL of modified YPDO medium (yeast extract 1% (w/v), peptone 2% (w/v), glucose 0.5% (w/v), MgSO_4_ 0.5% (w/v) and Tween 80 0.2% (v/v)) [32,33], during 120 h of fermentation and under the same conditions as the inoculum. Olive oil 5% (v/v) was used as an inductor at the beginning of the fermentation. All of the shake flasks for the preinoculum, inoculum and fermentation were sterilized at 121 °C for 15 min. The pH was controlled with phosphate buffer (0.1 M). One milliliter of sample was taken from the Erlenmeyer flasks every 12 h to be measured in the microplate and the SIA system.

### 2.3. Off-Line Lipase/Esterase Activity Determination

**Figure 1.**
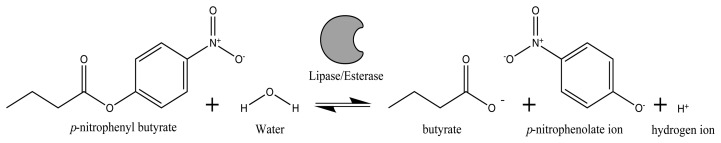
Enzymatic hydrolysis reaction of *p*-nitrophenyl butyrate.

Off-line lipase/esterase activity was determined spectrophotometrically using *p*-nitrophenyl butyrate (*p*-NPB) as the substrate. The release of the 4-nitrophenolate anion (yellow at pH values above its pKa = 7.08 at 22 °C) was monitored at *λ* = 415 nm during the enzymatic hydrolysis (Figure 1).

Enzymatic broth samples of *Yarrowia lipolytica* were taken out from the shake flasks cultures and centrifuged for 10 min, at 13,000 rpm and at 4 ^°^C. Supernatants were recovered for extracellular lipase/esterase activity determination. The reaction mixture was prepared by mixing one volume of a solution containing 10 mM of *p*-NPB into 2-methyl-2-butanol with 18 parts of a solution of phosphate buffer 50 mM, pH, 7.0% ± 0.5% (w/v) Triton X100. Ten microliters of the enzyme extract at an appropriate dilution and 190 (J.L of the reaction mixture were placed in a microplate of 96 wells. The release of the 4-nitrophenolate anion was monitored using a microplate reader Xmark, Bio-Rad (Hercules, CA, USA) at the following conditions: 30 °C, *λ* = 415 nm, during 10 min, and time intervals of 30 s [11,12]. All assays were performed in triplicate and represented as the mean value resulting from subtracting the kinetic of a given denatured sample under the same conditions and including the standard deviation. One enzyme unit (U) is defined as the amount of enzyme releasing one (j.mol of *p*-nitrophenol per minute under the tested conditions. Lipase activity (U/mL) is expressed as:

(1) $$\frac{U}{mL}=\frac{\Delta A-\Delta A_{\gamma }}{\in }\cdot \frac{\alpha }{\beta }\cdot \delta$$

where: Δ*A* = kinetic slope (Abs/min), ∈ = molar extinction coefficient (L/μmol;, *α*= reaction volume (L), *β*= sample volume (mL), *δ* = appropriate sample dilution and Δ*A_γ_* = kinetic without enzyme (U/mL).

### 2.4. Sequential Injection Analysis

In this section, the general structure of the lab-made SIA system is described.

#### 2.4.1. Hardware Description

**Figure 2.**
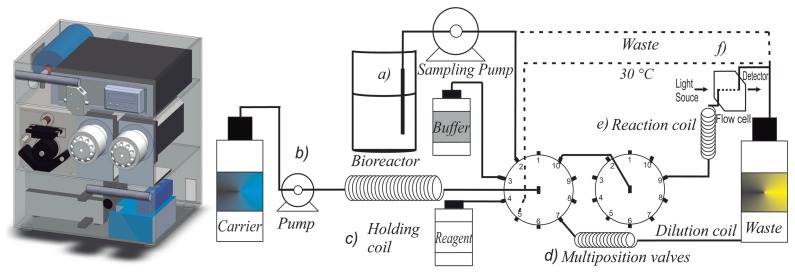
Description of the sequential injection analysis (SIA) system: **a:** sampling probe; **b:** mini-pump for aspiration/injection; **c:** holding coil; **d**: multiposition valves; **e**: reaction coil with temperature control; and **f**: flow cell with a light source and a spectrophotometer.

The SIA system was designed and built at the Industrial Biotechnology laboratory of the Centro de Investigación y Asistencia en Tecnología y Diseño del Estado de Jalisco A.C. The components of the SIA system are shown in Figure 2. The system includes a Watson-Marlow (Wilmington, MA, USA) laboratory mini pump (S2), a pair of ten-port multiposition valves, brand Valco Model C25-3180EMH-FL (Houston, TX, USA), an Ocean Optics USB-4000 spectrophotometer and a light source of tungsten-halogen Model HL-2000-LL (Dunedin, FL, USA). It also includes a flow cell FIA-Z-SMA-SS of stainless steel, a reaction coil FT-COIL90 with an integrated heater operating from room temperature to 90 °C, both Ocean Optics (Dunedin, FL, USA), a mixing chamber and a sampling probe of Applikon BioSenz of 385 mm (Heertjeslaan, The Netherlands). The tubing diameter is 0.8 mm i.dPTFE IDEX Health Science (Oak Harbor, WA, USA).

The SIA hardware components were driven by an assembled computer with Windows™ 7 home premium, an AMD Athlon™ X4 640 processor, 3.00 GHz, and 8 GB RAM. The Valco multiposition valves and the Watson-Marlow™ peristaltic pump were controlled via RS-232 serial ports. The spectrophotometer USB-4000 was enabled/disabled via a USB connection. The temperature reaction coil was driven with an ON-OFF controller Watlow™ EZ zone (Winona, MN, USA). The peristaltic pump for the sampling probe was controlled with an Arduino card with a USB connection.

**Figure 3.**
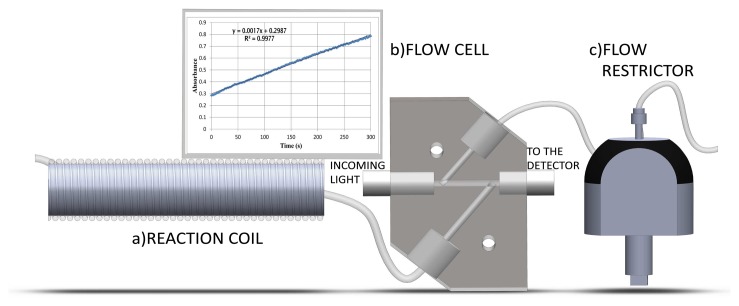
The components used to measure compounds in the SIA system: **a:** reaction coil; **b:** flow cell; and **c:** flow restrictor.

The main section in which the enzymatic reaction is measured is depicted in Figure 3. It is composed by a reaction coil with temperature control and a flow cell of stainless steel with stopped flow control. To avoid bubbles in the detector due to the carrier of the organic solvent mixture (*2*-methyl-2-butanol and phosphate buffer), a General Electric flow restrictor FR-902 (Pittsburgh, PA, USA) which pressurizes (0.2 MPa) the reaction coil and the flow cell, was included in the SIA system. The algorithm to control the hardware components will be described in the next section.

#### 2.4.2. Software Description

**Figure 4.**
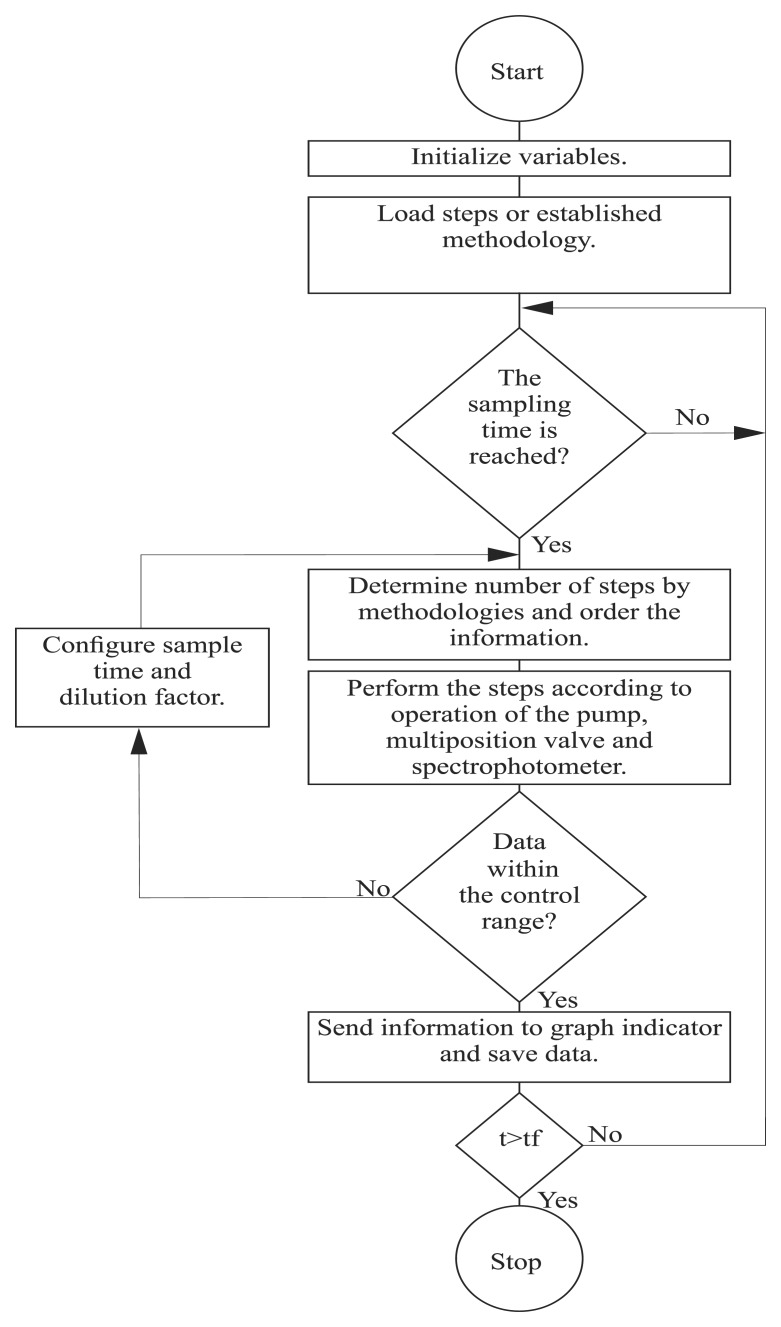
Operational flow diagram for the SIA system.

An algorithm to control the hardware components of the SIA system was designed with the software, LabVIEW™ v.11.0, National Instruments. Figure 4 shows the flow diagram of the algorithm to operate the SIA system. When the program is run for the first time, all of the variables, tables graphs and indicators are reset and cleared to store new information. The user can configure the method, i.e., aspiration and injection times, wavelength, *etc.*, directly with the software. In addition, when a method has been created previously, it can be saved and uploaded later for future use. When the sampling time is active, the software enables the multiposition valves and the peristaltic pump to mix the samples with the reactants according to the chosen methodology.

In addition, the set-point of the temperature controller and the value of the wavelength of the spectrophotometer can also be configured with the designed algorithm. When the analysis ends, the kinetic slope, the determination coefficient and the maximum value are calculated. This information is useful to determine if the result of the analysis is within a suitable detection range; if not, the designed software initializes the dilution sequence, and then, the sample is diluted, until it is within the robust region of detection. At the end of each cycle, the data are saved on the computer.

The algorithm inspects if the experimentation time was achieved. If the response is false, the software waits for the next sampling cycle; otherwise, the algorithm saves the data and finishes the sampling process. The designed software has a graphical interface containing five main screens: control and setting panels, status of the experimental kinetics, global process and additional methods. The diverse panels allow the user to configure the experimental and sampling time, to set detection limits and the sequence of operation, to enable the peristaltic pump and to configure the spectrophotometer, the automatic dilution and graph indicators, the dark field selection, the cleaning sequence, the open and saving file options, *etc.* In addition, the resulting spectra can be filtered to eliminate the schlieren effect [34]. The designed software can show the end point value or the resulting kinetics of using one or two wavelengths. The LabVIEW algorithm, which drives the spectrophotometer, was modified to include the Savitzky–Golay filter to smooth the generated signals [35].

#### 2.4.3. SIA Operating Sequence

The SIA operating sequence to determine lipase/esterase activity is shown in Table 1. The sequence is as follows: First, a cleaning procedure is carried out, where the excess volume of the holding coil is dispensed to the waste using distilled water as a carrier [36]. The analytical cycle begins aspirating the buffer into the holding coil, along with the substrate *p*-nitrophenyl butyrate dissolved in 2-methyl-2-butanol. The sequence follows the aspirating buffer. Later, the standard/sample is aspirated into the holding coil. To reduce the carrier influence in the reaction mixture, a small part of buffer is added. The mixture is taken to the reaction coil, and the temperature is set to 30 ^°^C. Later, the mixture is carried to the spectrophotometric detector; the flow is stopped, and the signal is read. The reaction kinetics is followed during 300 s. The differential absorbance measured at *λ*_1_ minus the one measured at *λ*_2_ is used, where *λ*_1_ = 415 nm and *λ*_2_ = 800 nm, in time intervals of 2 s. Finally, to avoid contamination from other residual substances, the carrier is injected. The results and discussion will be described in the next section.

## 3. Results and Discussion

### 3.1. Characterization of the SIA System

**Figure 5.**
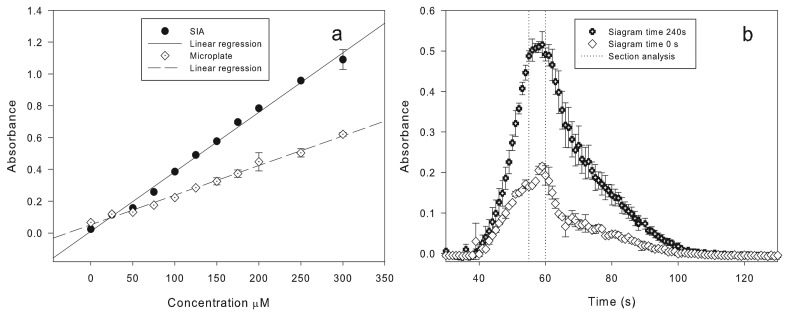
Determination of the molar extinction coefficient and “stopped flow” for the lipase/esterase activity using *p*-NPB as the substrate: (**a**) calibration curve of *p*-nitrophenol microplate Xmark vs. the SIA system; (**b**) “stopped flow” determination in two elapsed times of reaction, 0 s and 240 s.

Adapting the spectrophotometric microplate method using *p*-nitrophenyl butyrate as the substrate in the SIA system required characterizing the performance of the equipment, *i.e.*, determining the flows, operational times, injected volumes, valves sequence, *etc.* The dispersion factor is the relation of the concentration of a sample before and after it has been injected into the detector. The degree of dilution *D* of the sample caused by the SIA is given as:

(2) $$D=\frac{Co}{C}$$

where *Co* is the sample concentration before it is injected into the SIA system versus the maximum concentration of the sample *C* [22] in the transport process to the detector. To obtain D = 1, volumes greater than 585 μL at a rate of 14.6 μL/s should be injected into the system. A flow rate of 14.6 μL/s corresponds to one second of operation in the system with a standard deviation of 6%. To obtain the calibration curve of *p*-nitrophenol in the SIA, several concentrations of this chromophore were injected using a volume of 584 μL at different concentrations, *i.e.*, 50, 100, …, 300 μM. Samples were measured at *λ* = 415 nm. The achieved detection limit was 0–300 μm of *p*-nitrophenol corresponding to 0–1.2 absorbances in the spectrophotometer. Figure 5a shows the calibration curve of the microplate *versus* the SIA system. The determination coefficients obtained with the linear regression test for the off-line microplate method and the SIA system for the linear regression *y* = *ax* + *b* were (a = 3.74×10^−3^, b = 1.09×10^−2^; R^2^ = 0.99) and (a = 1.86×10^−3^, b = 5.18×10^−2^; R^2^ = 0.99), respectively.

The best mixing condition to get the maximum response in the SIA system at a flow rate of 14.6 μL/s was obtained injecting 9 s of buffer, 1 s of substrate, 9 s of buffer and 1 s of sample, and at the end of the sequence, 1 s of buffer was added to maintain the relation of the buffer-reaction mixture. These relationships allowed the diffusion and dispersion of the substrate in the sample buffer with a gradient concentration between the sample and the reagent, providing radial and axial dispersion, ensuring the maximum interpenetration of the analyte and the substrate [37,38].

During the experimentation, the presence of different refractive indices between the substrate and the reaction mixture was observed (schlieren effect) [34]. For this reason, the differential absorbance *λ* = *λ*_1_−*λ*_2_, where *λ*_1_ = 415 nm and *λ*_2_ = 800 nm, was used, and this way, the resulting absorbance is not affected by the schlieren effect. Figure 5b shows typical transient signals obtained from an SIA, named siagrams. Diverse siagrams for the lipase CaLB were performed in triplicate and expressed with standard deviation bars for the reaction mixture, during 0 s and 240 s of elapsed time of the reaction. From these experiments, the best mixing condition and stopping time were obtained. The maximum absorbance was reached at an injection time of 55–60 s in both experiments. To determine the measurement error between the SIA system and the off-line microplate spectrophotometric method, experimental kinetics were carried out at different stop times in the flow cell.

Table 2 shows the results of the kinetics with CaLB in the SIA system describing the stop time, activity, coefficient of determination (R^2^) of the kinetics, relative standard deviation (RSD; %) and RSD*_α_* (%), that is the standard deviation between the SIA and the off-line microplate spectrophotometric method. The minimum error between the off-line microplate spectrophotometric method and the SIA system was reached at the 56–57 s, obtaining an R^2^ of 0.98 with a difference of 4.3 and 5.9 RSD*_α_* (%), respectively. Up to five samples per hour with an RSD (%) of 4.29% can be analyzed with our SIA system [30]. The detection limit has a linear range between 0.05–1.60 U/mL and was calculated in triplicate. The system has the possibility to reach dilutions up to 1000 times during the analysis.

### 3.2. Monitoring Lipase/Esterase Activity during a Fermentation Process

**Figure 6.**
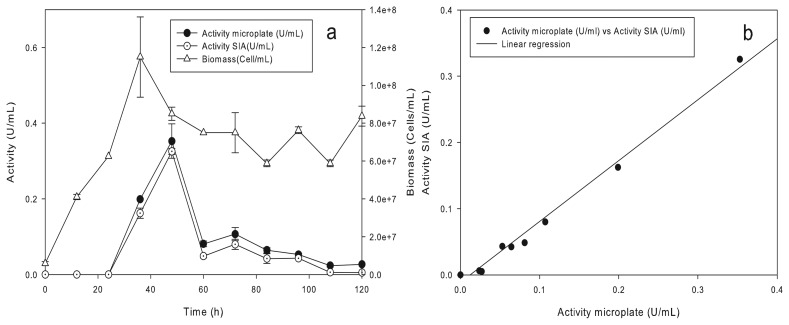
Monitoring the Yarrowia lipolytica fermentation with the SIA system. (a) The lipase/esterase activity obtained during the fermentation; (b) correlation obtained from the lipase/esterase activity in the off-line microplate method vs. the SIA method.

The SIA system should be configured and operated according to the sequences described in Table 2. Figure 6a shows the kinetics of growth and lipase production of the yeast *Yarrowia lipolytica* using *p*-nitrophenyl butyrate as the substrate and distilled water. The fermentation was monitored during 120 h, and samples were taken every 12 h, filtering samples off-line. In Figure 6a, it can be seen that the maximum biomass was achieved at 48 h of fermentation with 1.15·10^8^ cells/mL. The enzymatic activity was observed from the 36 h of the culture. The maximum value of 0.35 U/mL was achieved at 48 h and decreased almost completely at 108 h of fermentation, possibly due to the presence of proteases. The coefficients of determination obtained with the linear regression analysis *y = ax* + *b* resulted in the values: *a* = 0.92 and *b* = −0.01. The linear correlation between the hydrolytic activity obtained in the off-line microplate spectrophotometric method *versus* the SIA system is shown in Figure 6b. A *p*-value < 0.05 was obtained from an ANOVA analysis, showing the statistically significant relation between the activities obtained in both systems. The coefficient of determination indicates that the fitted model explains 98.92% of the variability in the activity of the SIA system.

An FIA system used to detect lipase activity was developed [17]. The method was based on determining the turbidity change of a triolein emulsion measured by a spectrophotometer at a fixed wavelength of *λ* = 340 nm (UV detection). A similar system designed to operate under the same wavelength using 1, 2-dioleoylglycerol as the substrate with a covariance of 2.2·10^−3^ U/mL was described [19]. The spectrum used in our lab-made SIA system can be configured from *λ* = 350 to 1000 nm. Additionally, our method operates in a visible wavelength *λ* = 415 nm, and can be implemented in common spectrophotometers. An FIA system coupled to an immobilized column with a commercial enzyme was used to detect glycerol and triacylglycerol in a fermentation process [18]. The potentiometric method, which is commonly used as a reference method to determine enzymatic activity, was coupled to an FIA system [20]. This system is not limited to a specific pH, increasing stability, improving the reproducibility and eliminating the schlieren effect. The disadvantages of potentiometric flow systems are the potential flow streams caused by the electrokinetic potential [22]. In addition, 15 min are required for an end point assay and 30 min for a kinetic reaction [20]. Our system has a spectrophotometric detector that is widely used to screen systems for the rapid analysis of samples. Although our system is limited to a specific pH, the use of a neutral pH helps in the study of a wide range of enzymes. The determination of the kinetic is about five minutes, and finally, the schlieren effect is reduced by using differential absorbance and the “stopped flow” approach. An SIA system with a non-specific substrate was used to detect lipase activity [30]. The system showed an adequate linear range of detection; although in a microorganism that produces an activity lower than 5 U/mL, it was not accurate. The use of SHC denotes higher precision and reproducibility on the kinetics determination. The proposed SIA system operates on enzyme activities higher than 0.05 U/mL. In addition, the rate reaction used in the flow cell of our system decreases the overestimation or underestimation of the enzymatic activity. Additionally, our SIA system can perform dilutions up to 1000 times and has a simple and easy to handle user interface.

## 4. Conclusions

An SIA analyzer for monitoring lipase/esterase activity using *p*-nitrophenyl butyrate as the substrate was developed. The lipase activity of a commercial enzyme, CaLB, on an SIA system was determined with RSD*_α_* 4.23%. The SIA system proved to be simple and robust at determining lipase activity in the range 0.05–1.60 U/mL, from off-line samples taken from shake flasks. It is possible to reach dilutions up to 1000 times, with an error around 8.75% DSR. In addition, the pressurized system and the implementation of the flow restrictor enhanced the mixer performance, avoiding bubble formation during the enzymatic reaction, which generates noise. The results are promising to monitor lipase activity in real time in a bioreactor, in which optimization and control strategies can be designed.

## Figures and Tables

**Table 1.** Operational sequence for the proposed SIA system used to determine lipase/esterase activity.

**Sequence**	**Pump Function**	Time * (s)	**Comment**
1	Injection	10	Cleaning the holding coil
2	Aspiration	9	Buffer aspiration into the holding coil
3	Aspiration	1	Substrate aspiration into the holding coil
4	Aspiration	9	Buffer aspiration into the the holding coil
5	Aspiration	1	Sample aspiration into the holding coil
6	Aspiration	1	Buffer aspiration into the holding coil
7	Injection	56	Mixture injection into the flow cell and stopped flow
8	Stopped flow	30	Flow stabilization and start the reaction
9	Stopped flow	300	Reading at *λ* = 415 nm in time intervals of 2 s.
10	Injection	50	Injection to waste

* The flow rate used was 14.6 μL/s in all steps.

**Table 2.** Results from the kinetics experiments for the off-line microplate spectrophotometric method and the SIA system.

**Stop Time (s)**	**SIA-activity (U/mL)**	**R^2^**	**RSD (%)**	${\text{RSD}}_{\alpha }^{*}(\%)$
55	982 ± 151	0.81	15.38	11.88
56	1,235 ± 108	0.98	8.75	4.29
57	1,069 ± 82	0.99	7.63	5.93
58	973 ± 89	0.99	9.11	12.56
59	942 ± 75	0.98	8.01	14.81
60	781± 164	0.99	21.0	27.79

* SIA-Microplate.
